# Association between CYP1A1 gene polymorphisms at rs4646903 and rs1048943 loci and susceptibility to prostate cancer: meta-analysis

**DOI:** 10.3389/fonc.2026.1779274

**Published:** 2026-04-15

**Authors:** Ruobing Teng, Peng Yu, Zhengqian Teng, Yujiang Feng

**Affiliations:** 1 Department of Urology, The Second Affiliated Hospital of Guilin Medical University, Guilin, Guangxi, China; 2 Department of Urology, The First Affiliated Hospital of Guilin Medical University, Guilin, Guangxi, China

**Keywords:** CYP1A1, CYP1A1 gene, gene polymorphism, meta-analysis, prostate cancer

## Abstract

**Objective:**

To evaluate the associations between C allele of CYP1A1 rs4646903 (T>C) and G allele of CYP1A1 rs1048943 (A>G) polymorphisms and prostate cancer (PCa) susceptibility under different genetic models.

**Methods:**

We systematically searched PubMed, EMBASE, CNKI, and Wanfang databases from inception to December 31, 2024. Eligible case–control studies were selected. Study quality was assessed using the Newcastle–Ottawa Scale (NOS). Meta-analyses were performed using RevMan 5.3 software.

**Results:**

Fifteen studies for rs4646903 and 14 studies for rs1048943 were included. For rs4646903 (T>C): The dominant model (CC+CT vs TT) showed that carrying the C allele was associated with increased PCa risk (OR = 1.39, 95%CI: 1.13–1.71, P = 0.002). For rs1048943 (A>G): Carrying the G allele was associated with decreased PCa risk in multiple models: allele model (G vs A: OR = 0.80, 95%CI: 0.71–0.90, P<0.001); homozygous model (GG vs AA: OR = 0.56, 95%CI: 0.42–0.76, P<0.001); dominant model (GA+GG vs AA: OR = 0.72, 95%CI: 0.55–0.94, P = 0.02); recessive model (GG vs GA+AA: OR = 0.78, 95%CI: 0.62–0.99, P = 0.04).

**Conclusion:**

The C allele of CYP1A1 rs4646903 may increase PCa risk under the dominant model. The G allele of CYP1A1 rs1048943 may reduce PCa risk across multiple genetic models.

## 1 Introduction

Prostate cancer (PCa) is the most common malignant tumor and the second leading cause of cancer-related death among men in Europe and the United States (1). In China, although PCa is historically considered a low-incidence disease, its incidence has increased rapidly in recent decades (2). The etiology of PCa is multifactorial and remains incompletely understood. Epidemiological evidence shows significant racial and geographic disparities: the incidence of PCa in Black populations is approximately 60-fold higher than in Chinese Han populations (3). Established risk factors include age, family history, ethnicity, and genetic predisposition (3, 4).

With the development of genomic research, single nucleotide polymorphisms (SNPs) have been recognized as the fundamental genetic basis of cancer susceptibility (4). Genome-wide association studies (GWAS) have identified more than 50 SNPs associated with PCa risk, and this number continues to grow with advances in sequencing technology (5).

Cytochrome P450 1A1 (CYP1A1) is a critical phase-I detoxification and metabolic enzyme encoded by the CYP1A1 gene, which is located on chromosome 15q22–24, with a total length of 5810 bp, including 7 exons and 6 introns. CYP1A1 participates in the metabolic activation of polycyclic aromatic hydrocarbons and other procarcinogens into electrophilic metabolites that form DNA adducts, leading to gene damage and malignant transformation, CYP1A1 is mainly expressed in the liver and is also detectable in prostate tissue (6, 7). Functional polymorphisms of CYP1A1 can alter gene expression, mRNA stability, or enzymatic activity, thereby affecting individual susceptibility to cancer. Among the well-studied variants, rs4646903 (T>C, also known as m3 site) is located in the 3'-flanking region, and rs1048943 (A>G, also known as m2 site or Ile462Val) is a missense variant in exon 7 that replaces isoleucine with valine, enhancing enzyme activity.

Numerous studies have explored the relationship between CYP1A1 rs4646903 and rs1048943 polymorphisms and PCa risk, but results remain inconsistent. Han (8) conducted a meta-analysis in 2013, but it included only a small number of studies, lacked comprehensive subgroup analyses, and did not cover newly published literature over the past decade. In addition, prior meta-analyses did not systematically evaluate both loci simultaneously, lacked strict quality control, and did not fully address heterogeneity or HWE deviations. Therefore, this updated meta-analysis was performed with expanded sample size, more rigorous methodology, complete subgroup analyses, and comprehensive quality and sensitivity assessments to clarify the precise associations of CYP1A1 rs4646903 and rs1048943 with PCa susceptibility.

## 2 Materials and methods

### 2.1 Literature search

This meta-analysis was performed in accordance with the Preferred Reporting Items for Systematic Reviews and Meta-Analyses (PRISMA) guidelines.

Databases: PubMed, EMBASE, CNKI, Wanfang. Time span: From inception to December 31, 2024.

Full search strings:

English: (CYP1A1 OR “Cytochrome P450 1A1”) AND (polymorphism OR variant OR SNP) AND (“prostate cancer” OR “prostate neoplasm”).

Chinese: (CYP1A1 OR 细胞色素 P450 1A1) AND (基因多态性 OR 变异) AND (前列腺癌 OR 前列腺肿瘤).

### 2.2 Inclusion and exclusion criteria

Inclusion criteria:

Study design: Case–control study.

Outcome: Association between CYP1A1 rs4646903 or rs1048943 and PCa.

Data availability: Genotype counts or allele frequencies in cases and controls.

Language: English or Chinese.

Exclusion criteria:

Studies without usable genotype data.

Duplicate publications based on the same cohort; only the most recent or complete study was retained.

Studies with NOS score < 5 stars (low quality).

### 2.3 Data extraction

Two reviewers independently screened titles, abstracts, and full texts. Discrepancies were resolved by a third reviewer. Extracted data included: first author, year, country, ethnicity, control source (population-based/hospital-based), genotyping method, genotype counts, and HWE P-value.

### 2.4 Quality assessment

Study quality was evaluated using the Newcastle–Ottawa Scale (NOS) (9). A score ≥ 6 stars was defined as high quality; 5 stars was moderate quality; < 5 stars was low quality and excluded.

### 2.5 Handling of duplicate cohorts

For multiple publications from the same study population, only the most recent and complete dataset was included to avoid duplication.

### 2.6 Consistency of allelic coding

All studies were uniformly coded with wild-type allele as reference to ensure consistency across analyses.

### 2.7 Statistical analysis

RevMan 5.3 was used. Heterogeneity was tested using the Q test and I² statistic. Heterogeneity: P > 0.05 and I² < 50% → fixed-effects model; Otherwise → random-effects model. Summary effects were reported as odds ratios (OR) and 95% confidence intervals (CI).Sensitivity analysis was conducted by sequentially omitting each study and by excluding HWE-deviated studies. Publication bias was evaluated using funnel plots.

## 3 Results

### 3.1 Retrieval results and basic characteristics of the included literature

**Figure 1:**
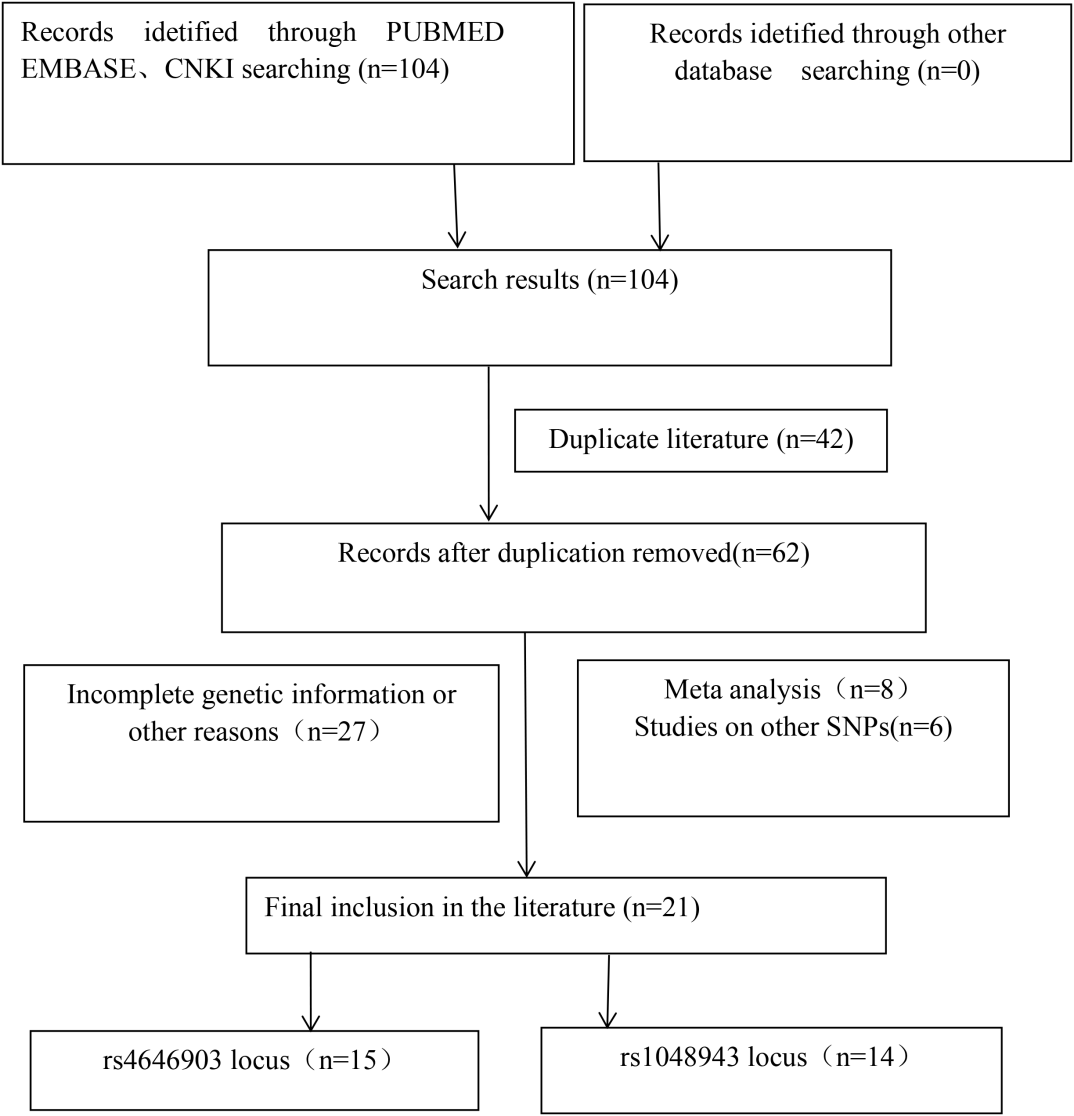
Literature search flow chart.

The flow chart of literature retrieval is depicted in **Figure 1** below. Initially, 104 articles were retrieved, and ultimately, 21 articles were included (10–30). There are 15 studies focusing on the CYP1A1 gene rs4646903 locus (T/C) gene polymorphism and prostate cancer (10–24), comprising 2,824 patients with prostate cancer and 2,663 control subjects. Additionally, 14 studies examine the rs1048943 (A/G) gene polymorphism and prostate cancer (11–13, 15–17, 20, 22, 25–30), encompassing 1,350 prostate cancer cases and 1,354 control cases. A genetic Hardy-Weinberg equilibrium (HWE) balance experiment was conducted on the control genotypes of all included studies. All control groups were tested for Hardy–Weinberg equilibrium (HWE). The basic characteristics of the included literature are detailed in **Table 1** below.

**Table 1:** Includes the basic features of the study.

Auther year	Country	Race	Control source	Genotyping methods	Sample(Case/Control)	Case			Control			HWE P- value	Quality evaluation
						AA	AB	BB	AA	AB	BB		
rs4646903 T>C													
Cristian (10) 2003	Chile	Mixed	H-B	PCR-RFLP	128/102	72	45	11	39	49	14	0.823	High
CHANG (11) 2003	America	Caucasian	P-B	PCR-RFLP	358/180	305	52	1	135	39	6	0.143	High
Gao (12) 2003	China	Asian	P-B	PCR-RFLP	58/112	16	33	9	36	61	15	0.172	High
Suzuki (13) 2003	Japan	Asian	P-B	PCR-RFLP	98/105	46	39	13	46	37	22	0.009	Low
Cacere (14) 2005	Chile	Mixed	P-B	PCR-RFLP	102/130	14	49	39	11	46	73	0.338	High
Guan (15) 2005	China	Asian	P-B	PCR-RFLP	83/115	31	40	12	51	42	22	0.018	Low
Vijayalakshmi (16) 2005	India	Caucasian	P-B	PCR-RFLP	50/50	30	19	1	44	6	0	0.652	High
Yang (17) 2006	China	Asian	H-B	PCR-RFLP	225/250	76	116	33	96	112	42	0.342	High
Quiñones (18) 2006	Chile	Mixed	P-B	PCR-RFLP	60/117	14	38	8	58	46	13	0.404	High
Mittal (19) 2007	India	Caucasian	P-B	PCR-RFLP	130/140	55	69	6	75	58	7	0.318	High
Li (20) 2008	China	Asian	P-B	PCR-RFLP	208/230	78	100	30	102	84	44	<0.01	Low
Lima (21) 2008	Brazil	Mixed	H-B	PCR-RFLP	125/100	94	26	5	69	27	4	0.516	High
Kumar (22) 2010	India	Caucasian	P-B	PCR-RFLP	70/71	18	31	21	16	31	24	0.33	High
Souiden (23) 2012	Tunisia	Caucasian	P-B	PCR-RFLP	138/138	111	24	3	114	21	3	0.105	High
Mittal (24) 2014	Kordia	Caucasian	H-B	PCR-RFLP	120/120	103	17	0	100	20	0	0.319	High
rs1048943 A>G													
Murata (25) 1998	Japan	Asian	H-B	PCR-RFLP	115/204	60	42	13	128	66	10	0.694	High
Murata (26) 2001	Japan	Asian	H-B	PCR-RFLP	115/200	60	42	13	125	64	11	0.462	High
Beer (27) 2002	America	Caucasian	P-B	PCR-RFLP	110/146	101	7	2	129	17	0	0.455	High
CHANG (11) 2003	America	Caucasian	P-B	PCR-RFLP	357/180	339	18	0	162	18	0	0.48	High
Gao (12) 2003	China	Asian	P-B	PCR-RFLP	58/112	15	31	12	42	59	11	0.136	High
Suzuki (13) 2003	Japan	Asian	P-B	PCR-RFLP	81/105	39	34	8	65	33	7	0.328	High
Guan (15) 2005	China	Asian	P-B	PCR-RFLP	83/115	48	30	5	75	33	7	0.207	High
Vijayalakshmi (16) 2005	India	Caucasian	P-B	PCR-RFLP	50/50	10	40	0	2	48	0	< 0.01	Low
Nock (28) 2006	America	Caucasian	P-B	PCR-RFLP	436/479	404	29	3	445	30	4	< 0.01	Low
Yang (17) 2006	China	Asian	H-B	PCR-RFLP	225/250	113	90	22	151	86	13	0.828	High
Li (20) 2008	China	Asian	P-B	PCR-RFLP	208/230	120	75	13	150	66	14	0.075	Low
Kumar (22) 2010	India	Caucasian	P-B	PCR-RFLP	70/61	16	31	23	14	29	18	0.725	High
Route (29) 2016	Iran	Caucasian	P-B	PCR-RFLP	79/79	25	47	7	44	23	12	< 0.01	Low
Sissung (30) 2016	America	Caucasian	P-B	PCR-RFLP	33/67	0	6	27	2	4	61	< 0.01	Low

HWE, Hardy-Winberg PCR-RFLP; polymerase chain reaction-restriction fragment length polymorphism H-B, hospital-based case–control study P-B, population-based case–control study.

### 3.2 Meta-analysis results

#### 3.2.1 rs4646903 locus (T/C)

**Figure 2:**
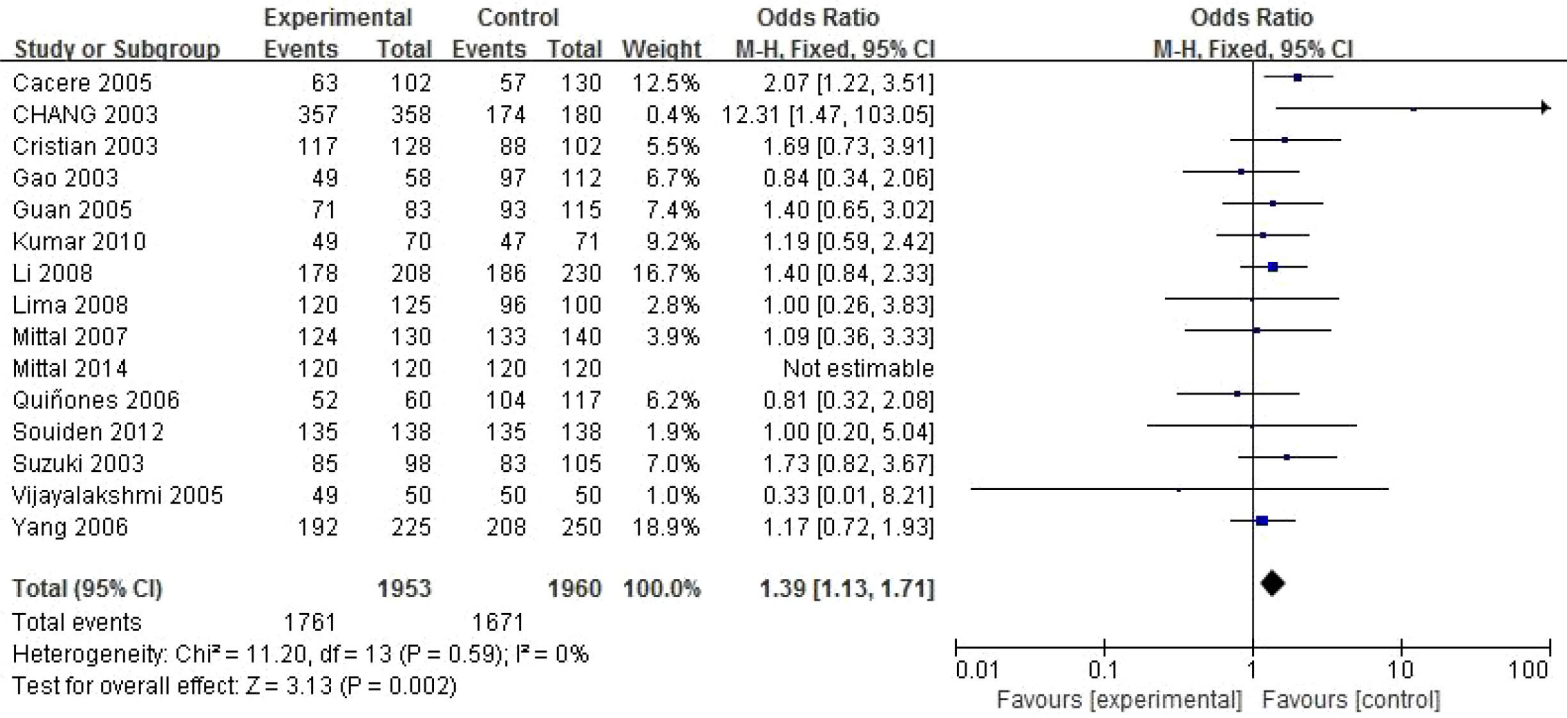
Forest map of rs4646903 locus SNPs and prostate cancer.

The meta-analysis results detailing the correlation between the CYP1A1 gene rs4646903 polymorphism and prostate cancer are presented in **Table 2**. Significant association was only observed in the dominant model (CC+CT vs TT):OR=1.39, 95%CI=1.13–1.71, P = 0.002, I²=0%.Other models showed no significant association. Subgroup analysis revealed that the association varied by ethnicity and control source: Mixed ethnicity showed increased risk. In Asians, no significant increased risk was observed. The dominant model forest map is shown in **Figure 2** below, And the remaining gene model forest maps are added to the **Supplementary Materials**.

**Table 2:** Meta-analysis results of rs4646903 locus SNPs and prostate cancer susceptibility.

Stratification	C vs T				CC vs TT			
	OR(95%CI)	P	Ph	I^2^	OR(95%CI)	P	Ph	I2
All	1.05[0.86;1.27]	0.65	<0.01	69%	1.21[0.90;1.62]	0.21	0.18	25%
Race								
Asian	0.98[0.84;1.14]	0.79	0.76	0%	1.11[0.81;1.51]	0.51	0.77	0%
Caucasus	0.95[0.61;1.50]	0.84	<0.01	78%	1.32[0.56;3.12]	0.53	0.16	38%
Mix	1.20[0.71;2.04]	0.5	<0.01	83%	1.30[0.55;3.07]	0.54	0.04	64%
Control source								
P-B	0.98[0.76;1.26]	0.86	<0.01	74%	1.17[0.81;1.70]	0.41	0.14	32%
H-B	1.22[0.90;1.60]	0.19	0.11	50%	1.31[0.76;2.26]	0.33	0.27	23%
CT vs TT					CT+CC vs T			
	OR(95%CI)	P	Ph	I2	OR(95%CI)	P	Ph	I2
All	0.86[0.66;1.13]	0.28	<0.01	67%	1.39[1.13;1.71]	0	0.59	0%
Race								
Asian	0.73[0.59;0.92]	0.01	0.84	0%	1.30[0.98;1.72]	0.07	0.78	0%
Caucasus	0.86[0.52;1.42	0.55	<0.01	72%	1.34[0.66;2.71]	0.42	0.25	25%
Mix	1.01[0.44;2.34]	0.98	<0.01	83%	1.55[1.01;2.37]	0.04	0.34	11%
Control source								
P-B	0.74[0.52;1.02]	0.07	<0.01	64%	1.41[1.10;1.82]	0.01	0.41	3%
H-B	1.42[0.78;1.97]	0.37	<0.01	64%	1.26[0.84;1.90]	0.26	0.72	0%
CC vs CT+TT								
	OR(95%CI)	P	Ph	I2				
All	0.93[0.71;1.22]	0.61	<0.01	70%				
Race								
Asian	0.83[0.67;1.02]	0.08	0.81	0%				
Caucasus	0.88[0.52;1.51]	0.65	<0.01	77%				
Mix	1.12[0.49;2.55]	0.79	<0.01	85%				
Control source								
P-B	0.82[0.59;1.15]	0.25	<0.01	71%				
H-B	1.26[0.81;1.79]	0.3	<0.01	64%				

H-B, hospital-based case–control study; P-B, population-based case–control study.

#### 3.2.2 rs1048943 (A/G)

The meta-analysis results regarding the correlation between the CYP1A1 gene rs1048943 polymorphism and prostate cancer are presented in **Table 3**. Carrying the G allele was significantly associated with reduced PCa risk in: Allele model (G vs A): OR = 0.80;Homozygous model (GG vs AA): OR = 0.56;Dominant model: OR = 0.72.

**Table 3:** Meta-analysis results of rs1048943 locus SNP and prostate cancer susceptibility.

Stratification	G vs A				GG vs AA			
	OR(95%CI)	P	Ph	I^2^	OR(95%CI)	P	Ph	I2
All	0.82[0.70;0.96]	0.01	0.9	35%	0.56[0.42;0.76]	<0.01	0.71	0%
Race								
Asian	0.71[0.61;0.81]	<0.01	0.93	0%	0.50[0.35;0.70]	<0.01	0.64	0%
Caucasus	1.09[0.83;1.44]	0.54	0.17	34%	0.84[0.46;1.54]	0.57	0.81	0%
Mix								
Control source	0.90[0.74;1.09]	0.01	0.11	39%	0.07[0.48;1.03]	0.07	0.78	0%
P-B	0.69[0.57;0.83]	<0.01	0.77	0%	0.41[0.42;0.76]	<0.01	0.94	0%
GA vs AA					GA+GG vs AA			
	OR(95%CI)	P	Ph	I2	OR(95%CI)	P	Ph	I2
All	0.91[0.61;1.35]	0.46	0.06	42%	0.73[0.53;1.01]	0.06	0.2	25%
Race								
Asian	0.70[0.50;0.99]	0.05	0.62	0%	0.57[0.41;0.79]	<0.01	0.65	0%
Caucasus	1.49[0.60;3.66]	0.39	0.06	56%	1.22[0.72;2.09]	0.46	0.36	9%
Mix								
Control source	1.15[0.70;1.89]	0.57	0.09	41%	0.92[0.64;1.33]	0.66	0.34	11%
P-B	0.56[0.35;0.91]	0.02	0.93	0%	0.46[0.29;0.73]	<0.01	0.72	0%
CC vs CT+TT								
	OR(95%CI)	P	Ph	I2				
All	0.78[0.62;0.99]	0.04	<0.01	51%				
Race								
Asian	0.66[0.56;0.79]	<0.01	0.99	0%				
Caucasus	1.14[0.63;2.05]	0.66	<0.01	70%				
Mix								
Control source	0.87[0.62;1.21]	0.4	<0.01	60%				
P-B	0.66[0.51;0.84]	<0.01	0.99	0%				

H-B, hospital-based case–control study; P-B, population-based case–control study.

**Figure 3:**
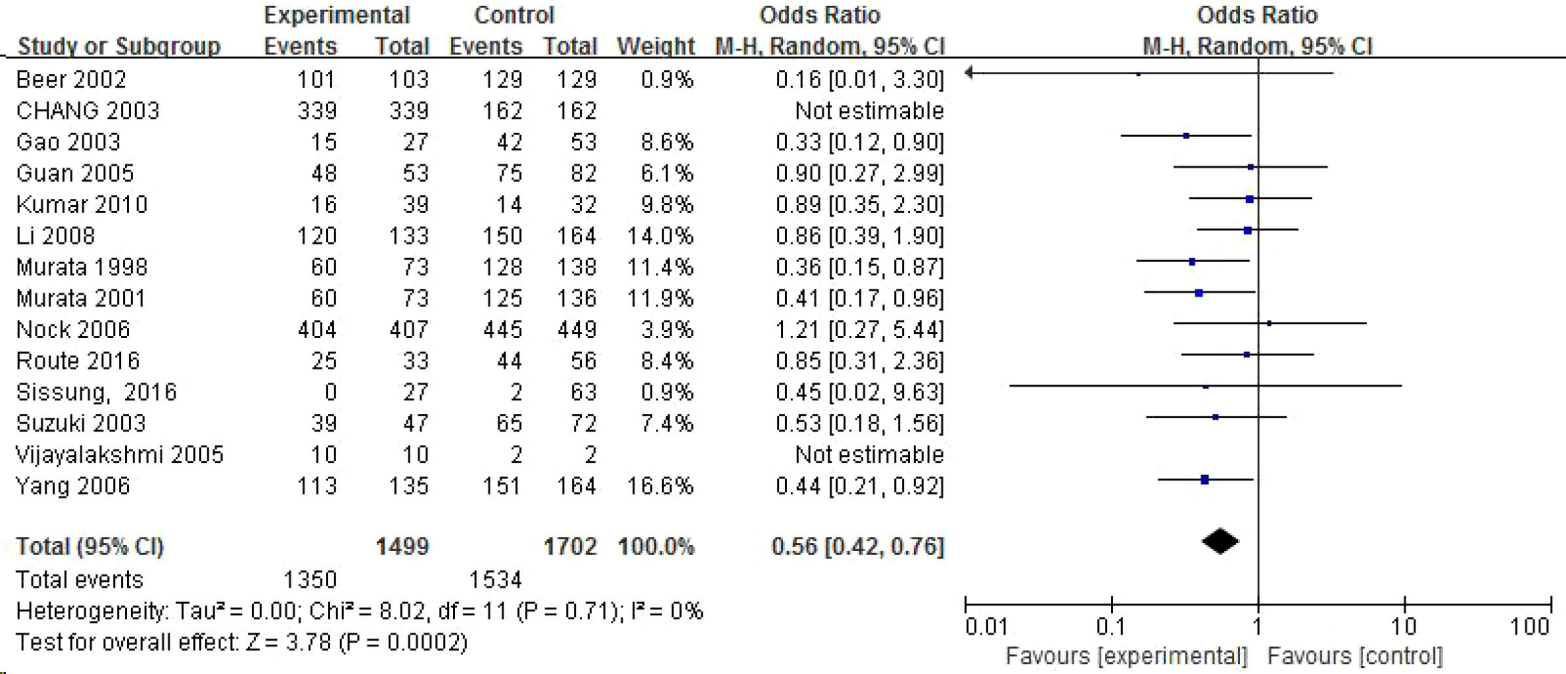
Forest map of rs1048943 locus SNPs and prostate cancer (homozygous model).

Recessive model: OR = 0.78;Subgroup analysis confirmed a strong protective effect in Asian populations, but not in Caucasians. The homozygous model forest plot is illustrated in **Figure 3** below, whereas the forest plots for the remaining gene models are added to the **Supplementary Materials**.

### 3.3 Sensitivity analysis

The sensitivity analysis of rs4646903 SNPs and their association with prostate cancer susceptibility revealed that the HWE test results of the included studies indicated that the studies by Suzuki (2003) (13), Guan (2005) (15), and Li (2008) (20) did not conform to the Hardy-Weinberg equilibrium (HWE). However, the results of the meta-analysis did not change significantly after excluding any one or a combination of these three studies. Furthermore, upon sequentially removing each of the included studies and re-performing the meta-analysis, the results remained close to the original odds ratio (OR) values, suggesting that the findings of this study are relatively stable. Similarly, a meta-analysis of SNPs in CYP1A1 rs1048943 did not identify any single study that had a significant impact on the overall effect size.

### 3.4 Publication bias

**Figure 4:**
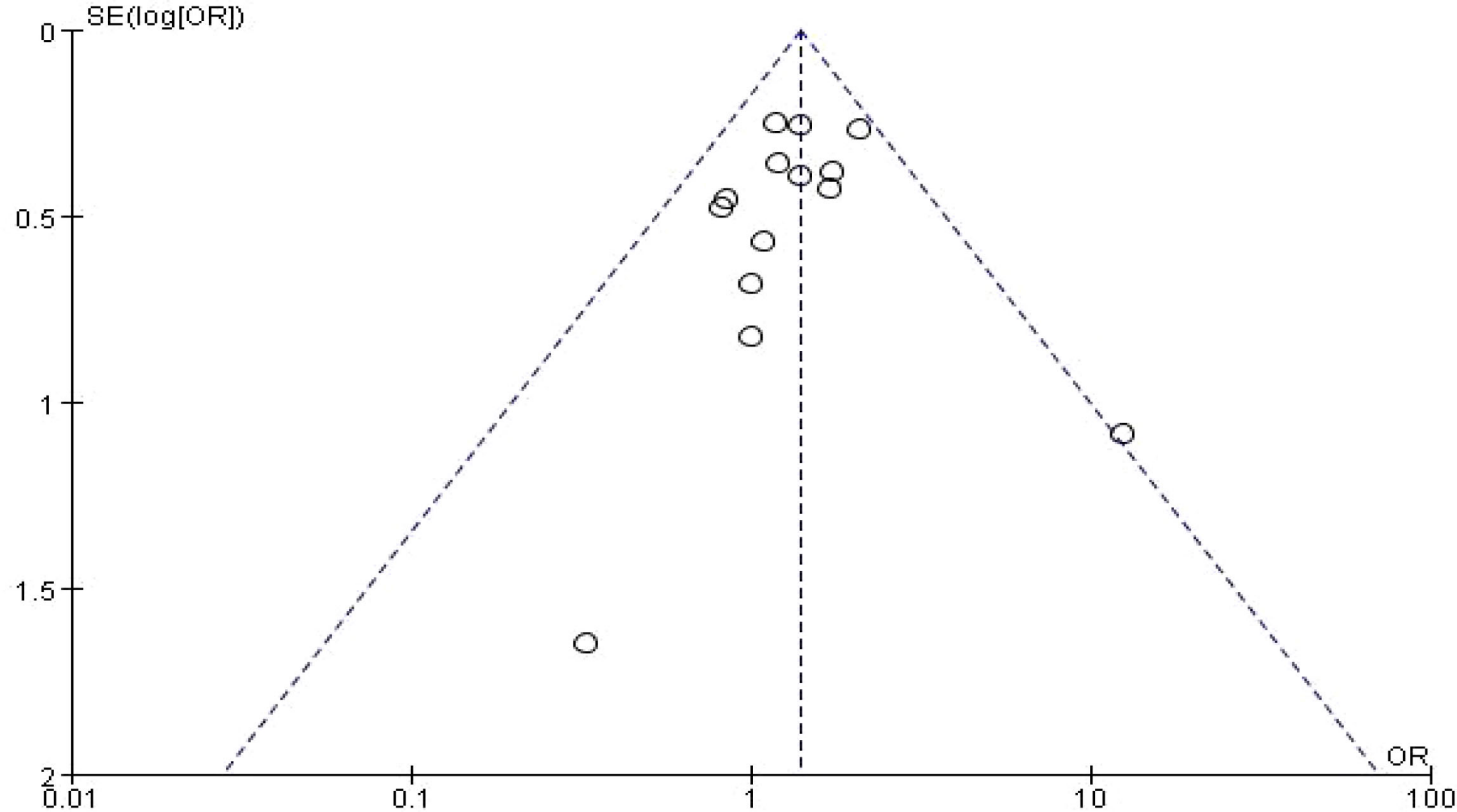
Funnel diagram of rs4646903 locus SNPs and prostate cancer dominant model.

**Figure 5:**
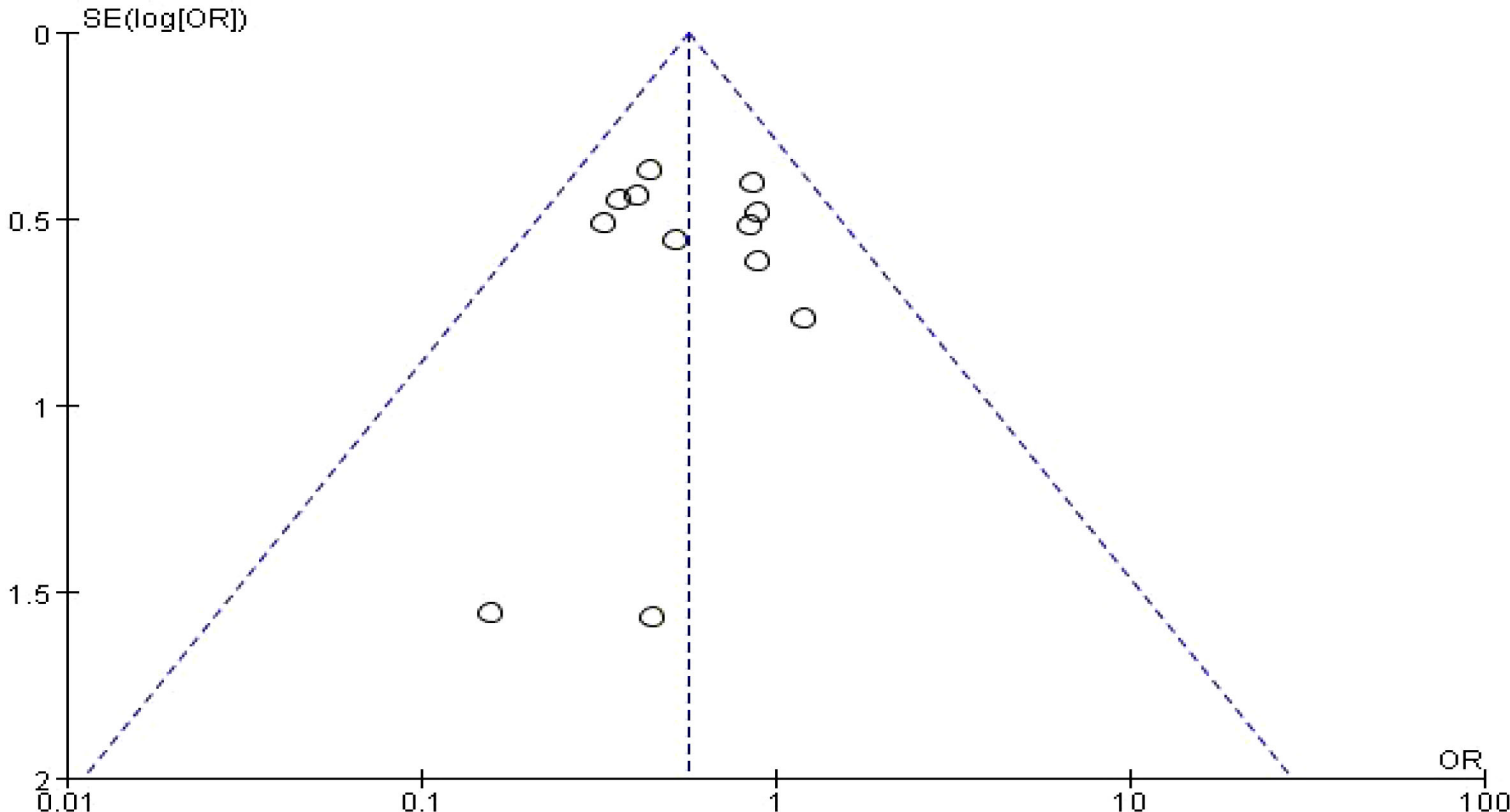
Funnel plot of rs1048943 locus SNPs and prostate cancer homozygous model.

No publication bias was detected in the study of rs4646903 SNPs and their association with prostate cancer susceptibility. The funnel plot for the dominant model (TC + CC vs. TT) is presented in **Figure 4** below. Additionally, no publication bias was observed in the study of the rs1048943 SNP and prostate cancer susceptibility. The funnel plot for the homozygous model (GG vs. AA) is shown in **Figure 5** below. The funnel plots for other gene models are added to the **Supplementary Materials**.

## 3 Discussion

Cytochrome P450 is a group of isoenzymes encoded by structurally and functionally related superfamily genes. CYP1A1 is widely distributed in lung, kidney, gastrointestinal tract, skin, larynx, placenta, lymphocytes, brain, and other extrahepatic tissues. It accounts for 2.5% in the metabolism of exogenous substances and participates in the metabolism of hydrocarbon carcinogens. CYP1A1 rs4646903 and rs1048943 SNPs are associated with the occurrence of many tumors. Nie et al. (31) reported that the CYP1A1 locus SNP can be used as a predictor of non-small cell lung cancer. Oztürk et al. (32) reported that the CYP1A1 rs1048943 SNP is a risk factor for bladder cancer susceptibility in Turkish people. Additionally, there is a relationship with ovarian cancer (33), breast cancer (34), and other tumors. There are often many reports with conflicting conclusions on the same tumor. Consequently, some meta-analyses have further examined the relationship between SNPs at various sites of CYP1A1 and tumor susceptibility by establishing inclusion criteria and expanding the sample size. Although there are other sporadic meta-analysis reports, Our study included more additional recent studies, Simultaneously analyzed two loci, and performed complete ethnicity and control-source subgroups, Quantitatively assessed HWE deviation and sensitivity. Our study was to investigate the correlation between CYP1A1 rs4646903 and rs1048943 SNPs and prostate cancer susceptibility.

The present meta-analysis demonstrated that the C allele of CYP1A1 rs4646903 was associated with an increased risk of prostate cancer under the dominant model (CC+CT vs TT) (OR = 1.39, 95%CI: 1.13–1.71, P = 0.002). For CYP1A1 rs1048943, the G allele was significantly associated with decreased prostate cancer risk across multiple genetic models, including the allele model, homozygous model, dominant model, and recessive model. These results suggest that the rs4646903 C allele may be a potential risk factor, while the rs1048943 G allele may serve as a protective factor for prostate cancer. Biologically, the rs4646903 polymorphism is located in the 3′-flanking region and may affect gene transcription and expression efficiency, thereby enhancing the activation of carcinogens and increasing cancer susceptibility. In contrast, the rs1048943 polymorphism causes an amino acid substitution from isoleucine to valine, which alters enzyme activity. Although this variant increases catalytic activity, it may accelerate the detoxification of harmful metabolites, thereby exerting a protective effect against prostate cancer. These mechanisms may partially explain the opposite effects of the two polymorphisms observed in this study.

### 3.1 Heterogeneity and methodological considerations

Significant between-study heterogeneity was observed in several genetic models for rs4646903 (I²=67%–70%). Potential sources of heterogeneity include differences in ethnicity, publication year, sample size, genotyping methods, control source (population-based vs. hospital-based), and varying adjustment for confounding factors such as age, smoking status, and dietary habits across studies. Notably, the dominant model for rs4646903 showed no heterogeneity (I²=0%), which may be attributed to the fact that this model combines heterozygous and homozygous mutant genotypes, thereby reducing inter-study variability and yielding more consistent effect estimates across included populations. Regarding multiple comparisons, a total of four genetic models were analyzed for each polymorphism, and further subgroup analyses were conducted by ethnicity and control source. These subgroup analyses were exploratory in nature to identify potential effect modifiers rather than confirmatory analyses. Accordingly, Bonferroni or false discovery rate (FDR) corrections for multiple testing were not applied, consistent with conventional reporting practices in meta-analyses of genetic association studies. All results should be interpreted with the exploratory nature of subgroup analyses in mind. Subgroup analyses revealed that the association of rs4646903 with prostate cancer risk varied by ethnicity. In Asian populations, the variant allele did not increase cancer risk, whereas in mixed populations, a significant risk-increasing effect was observed. For rs1048943, the protective effect was particularly strong in Asian populations but not evident in Caucasian populations. These ethnic differences may be caused by genetic background, environmental exposure, lifestyle factors, and gene-environment interactions. Previous scholars (30) included eleven case-control studies in their meta-analysis. The results indicated that CYP1A1 rs4646903 SNPs increased susceptibility to prostate cancer. SNPs of rs1048943 were not associated with prostate cancer susceptibility. Considering the limited number of documents included, the overall quality of the literature is low. The present study includes a larger number of studies, longer search duration, more comprehensive subgroup analyses, and more rigorous quality control. The previous study included fewer articles and lacked detailed assessments of heterogeneity, HWE deviation, and sensitivity. Our updated results are therefore more reliable and comprehensive.

Although the literature search was updated to December 31, 2024, no new eligible case-control studies were found after 2016. This is mainly because the focus of prostate cancer genetic research has shifted from candidate-gene polymorphism studies to genome-wide association studies (GWAS), large consortia such as PRACTICAL, and polygenic risk score analysis. Consequently, very few new candidate-gene studies on CYP1A1 polymorphisms have been published in recent years.

Several included studies were not in Hardy-Weinberg equilibrium and were rated as low quality. However, sensitivity analyses excluding all HWE-deviated and low-quality studies showed that the pooled effect sizes did not change significantly, supporting the stability and reliability of the main conclusions. Retaining these studies in the primary analysis avoided selection bias and ensured the comprehensiveness of the meta-analysis.

The major strengths of this meta-analysis include: (1) the largest sample size so far for both CYP1A1 rs4646903 and rs1048943; (2) strict inclusion and exclusion criteria and quality evaluation; (3) comprehensive subgroup analyses; (4) complete sensitivity analysis and publication bias assessment; (5) clear specification of allele-specific and model-specific effects; and (6) consistent allelic coding across all studies.

Several limitations should also be acknowledged. First, only English and Chinese literature was included, and only PubMed, EMBASE, and CNKI were searched; relevant studies may exist in other databases such as Web of Science and Scopus. Second, lack of individual participant data limited further adjustment for confounding factors. Third, moderate to high heterogeneity existed in some models. Fourth, all subgroup analyses were exploratory without correction for multiple testing. Finally, the number of studies in some ethnic subgroups was relatively small.

In conclusion, the CYP1A1 rs4646903 C allele is associated with increased prostate cancer risk under the dominant model, while the CYP1A1 rs1048943 G allele is associated with decreased prostate cancer risk. These associations are ethnicity-specific and model-specific. These findings provide evidence for understanding the genetic susceptibility of prostate cancer. Further large-scale, well-designed prospective studies are warranted to verify these results.

## Data Availability

The original contributions presented in this study are included in the article and its **Supplementary Materials**. Further inquiries regarding the data can be directed to the corresponding authors (mryupeng@outlook.com; 2263422429@qq.com).
