# Supplementary figures and images for: Association between CYP1A1 gene polymorphisms at rs4646903 and rs1048943 loci and susceptibility to prostate cancer: meta-analysis
Source: Front Oncol. 2026 Apr 15;16:1779274. doi: 10.3389/fonc.2026.1779274 (PMC13124557; doi:10.3389/fonc.2026.1779274)

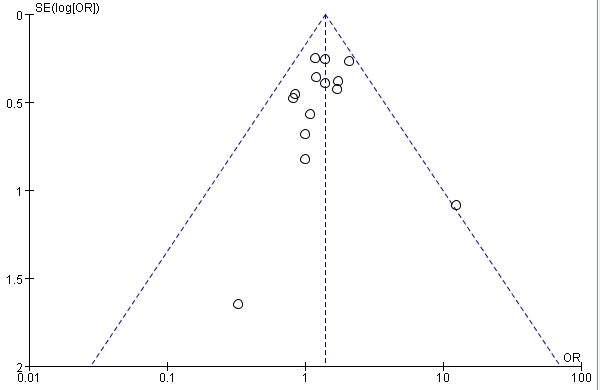

Supplement: Supplementary file 1 [file DataSheet1.zip › rs4646903 图片/共显性模型 CC+CT VS TT/漏斗图.jpg]

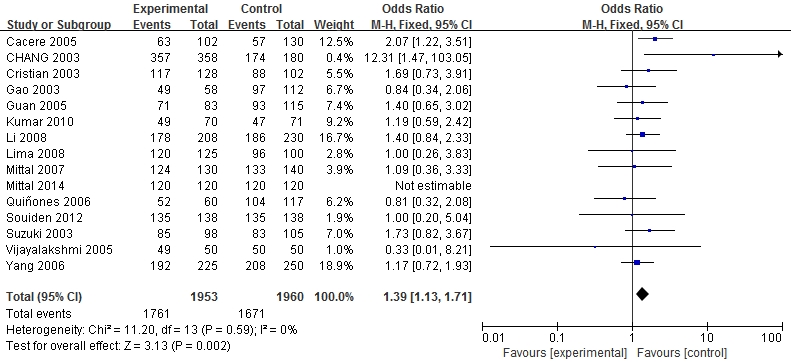

Supplement: Supplementary file 1 [file DataSheet1.zip › rs4646903 图片/共显性模型 CC+CT VS TT/线性模型 森林图.jpg]

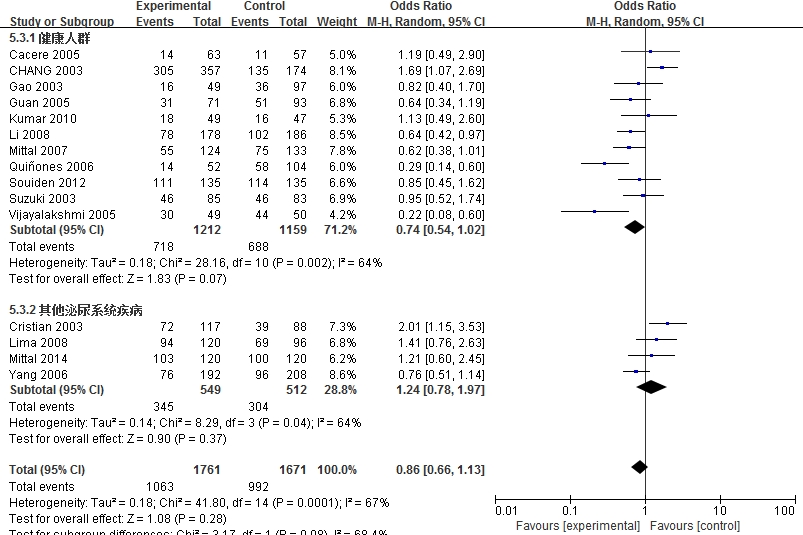

Supplement: Supplementary file 1 [file DataSheet1.zip › rs4646903 图片/杂合模型 CT vs TT/亚组分析 对照组来源.jpg]

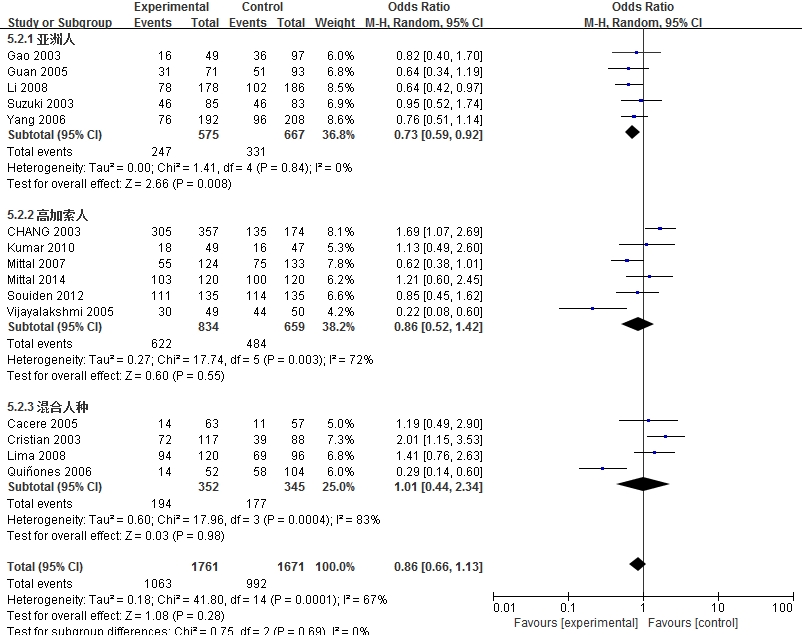

Supplement: Supplementary file 1 [file DataSheet1.zip › rs4646903 图片/杂合模型 CT vs TT/亚组分析 种族.jpg]

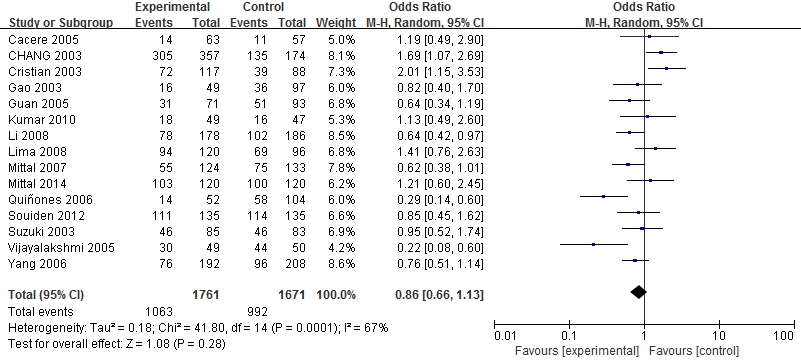

Supplement: Supplementary file 1 [file DataSheet1.zip › rs4646903 图片/杂合模型 CT vs TT/森林图.jpg]

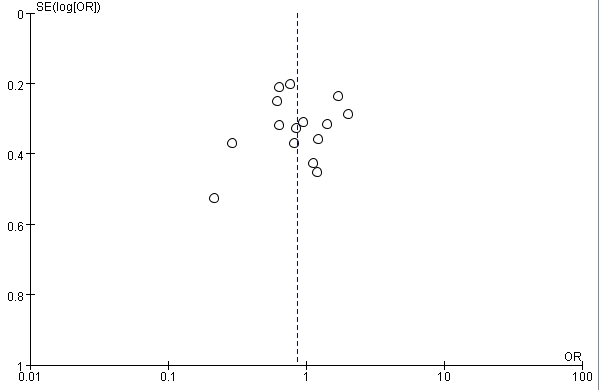

Supplement: Supplementary file 1 [file DataSheet1.zip › rs4646903 图片/杂合模型 CT vs TT/漏斗图.jpg]

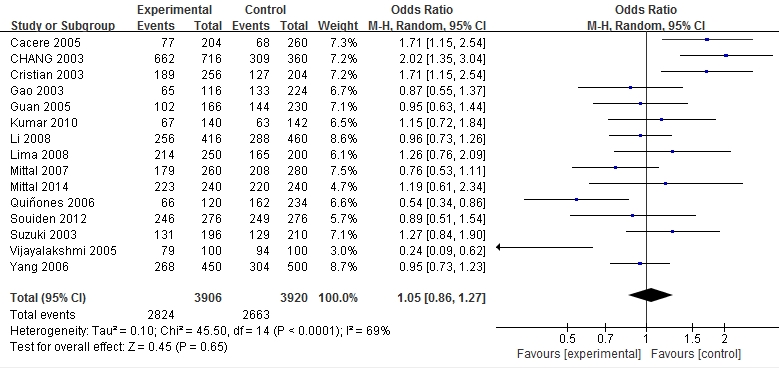

Supplement: Supplementary file 1 [file DataSheet1.zip › rs4646903 图片/等位基因模型 C vs T/cyp1a1 rs4646903 等位基因模型 森林图.jpg]

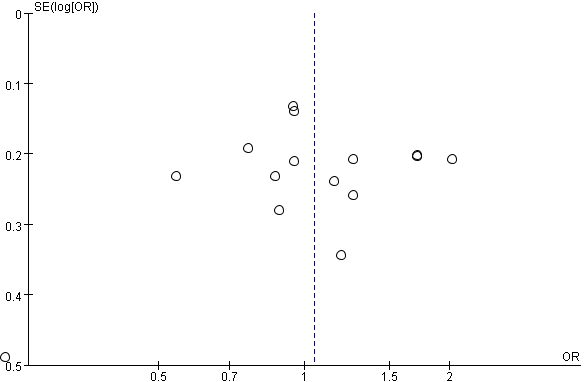

Supplement: Supplementary file 1 [file DataSheet1.zip › rs4646903 图片/等位基因模型 C vs T/cyp1a1 rs4646903 等位基因模型 漏斗图.jpg]

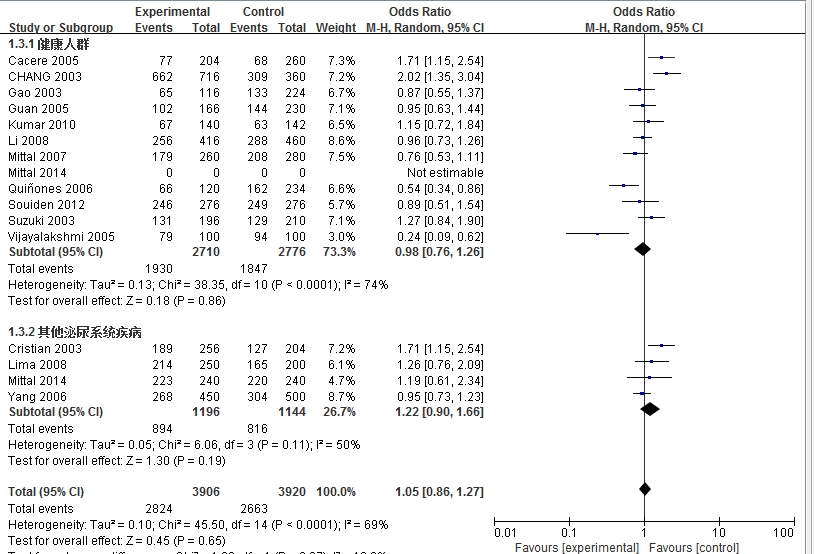

Supplement: Supplementary file 1 [file DataSheet1.zip › rs4646903 图片/等位基因模型 C vs T/根据对照组来源进行亚组分析.jpg]

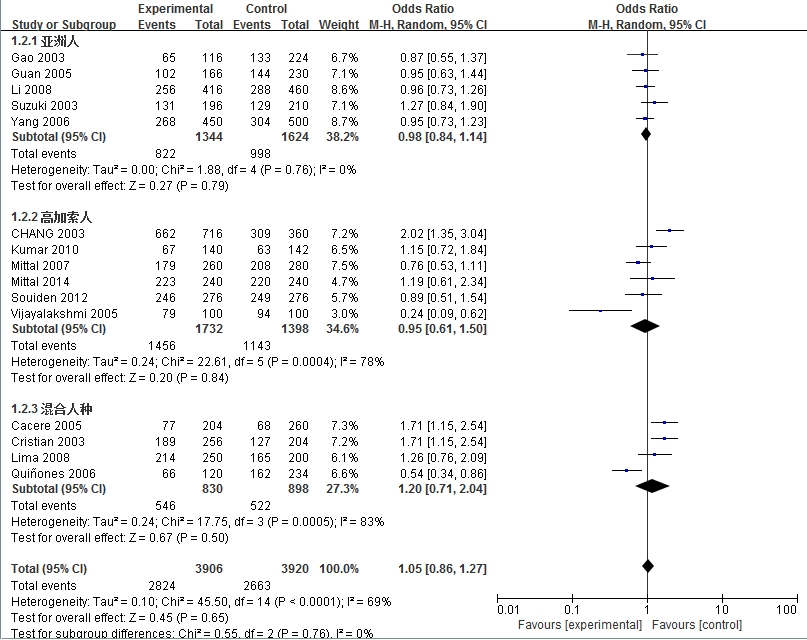

Supplement: Supplementary file 1 [file DataSheet1.zip › rs4646903 图片/等位基因模型 C vs T/根据种族进行亚组分析.jpg]

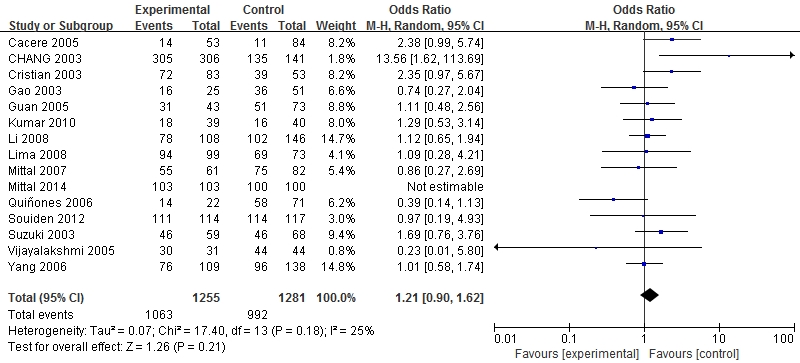

Supplement: Supplementary file 1 [file DataSheet1.zip › rs4646903 图片/纯合模型 CC VS TT/森林图.jpg]

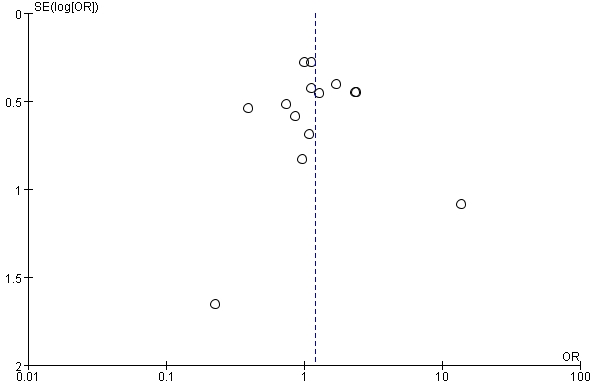

Supplement: Supplementary file 1 [file DataSheet1.zip › rs4646903 图片/纯合模型 CC VS TT/漏斗图.jpg]

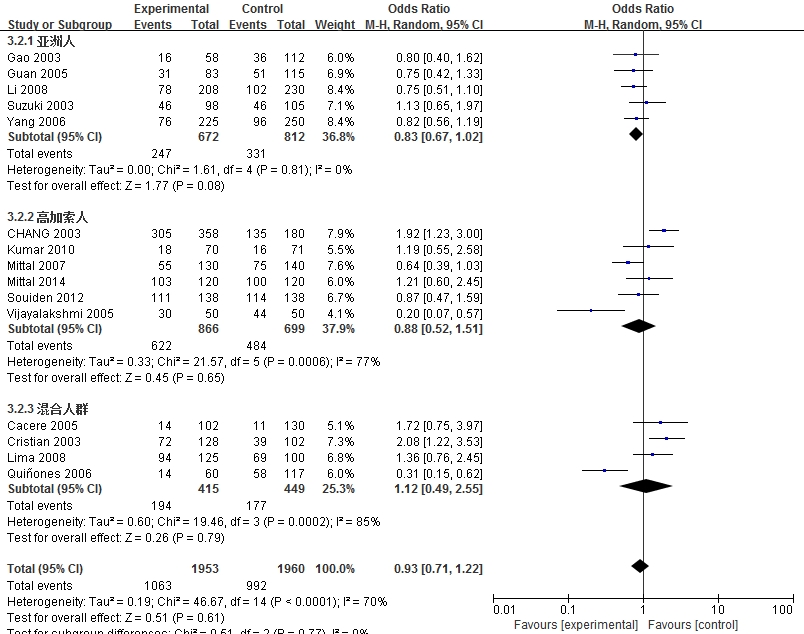

Supplement: Supplementary file 1 [file DataSheet1.zip › rs4646903 图片/隐形模型 CC vs CT+TT/亚组分析 森林图.jpg]

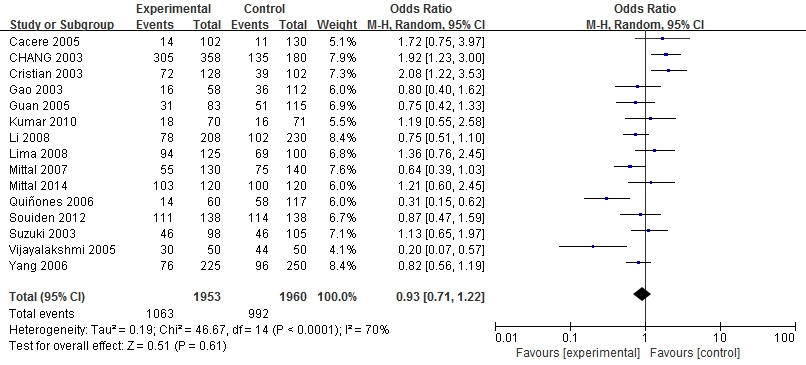

Supplement: Supplementary file 1 [file DataSheet1.zip › rs4646903 图片/隐形模型 CC vs CT+TT/森林图.jpg]

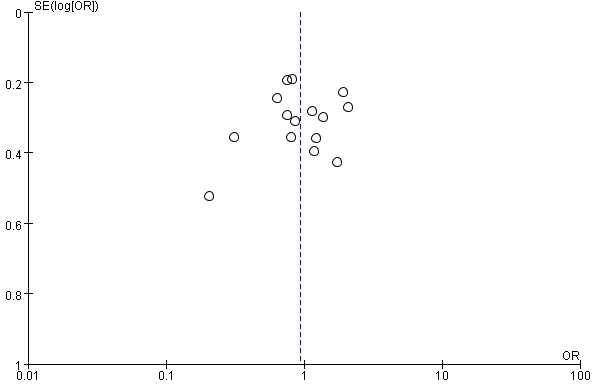

Supplement: Supplementary file 1 [file DataSheet1.zip › rs4646903 图片/隐形模型 CC vs CT+TT/漏斗图.jpg]

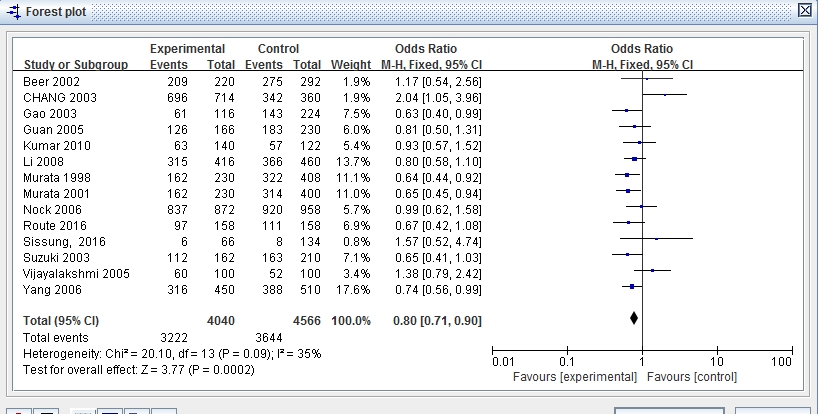

Supplement: Supplementary file 2 [file DataSheet2.zip › rs1048943/Avs G.jpg]

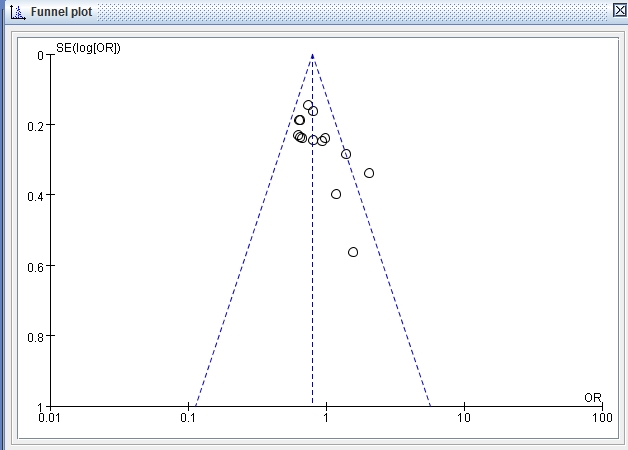

Supplement: Supplementary file 2 [file DataSheet2.zip › rs1048943/AvsG 漏斗图.jpg]

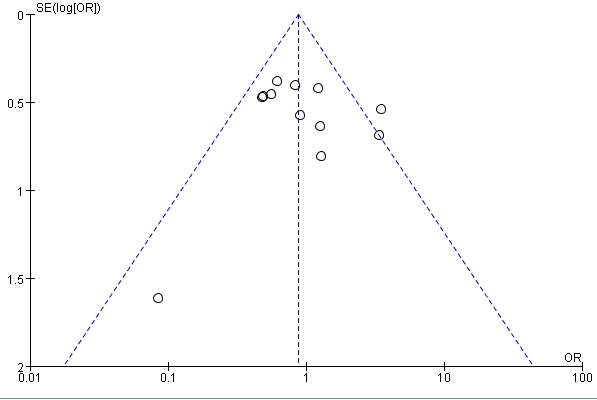

Supplement: Supplementary file 2 [file DataSheet2.zip › rs1048943/GA VS AA 漏斗图.jpg]

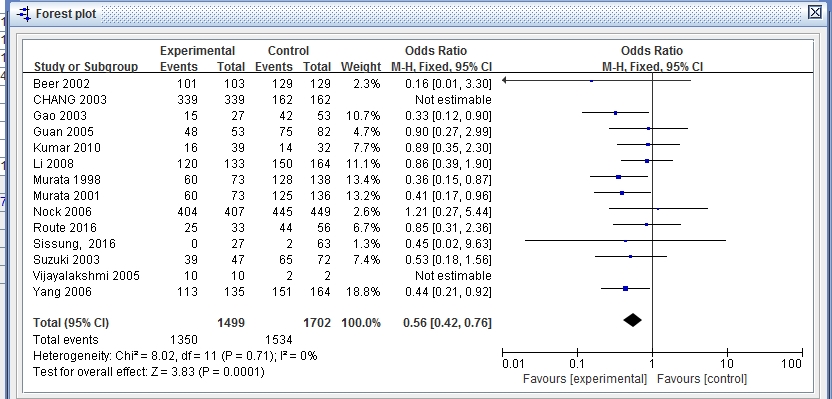

Supplement: Supplementary file 2 [file DataSheet2.zip › rs1048943/GG vs AA 森林图.jpg]

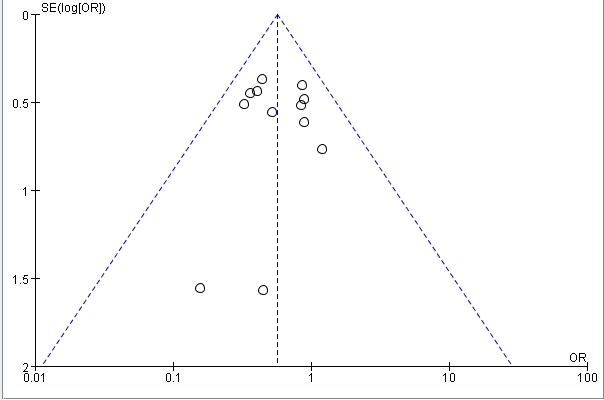

Supplement: Supplementary file 2 [file DataSheet2.zip › rs1048943/GG vs AA漏斗图.jpg]

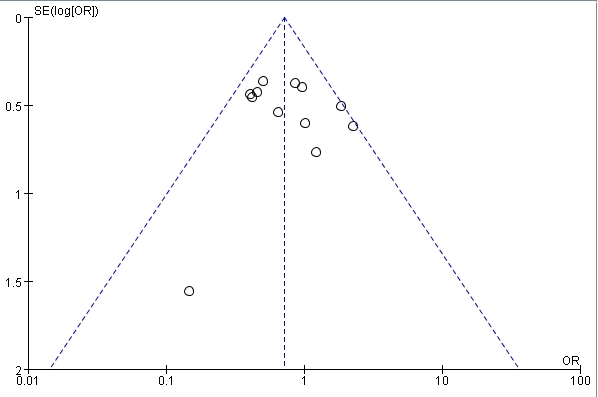

Supplement: Supplementary file 2 [file DataSheet2.zip › rs1048943/GG+GA VS AA 漏斗图.jpg]

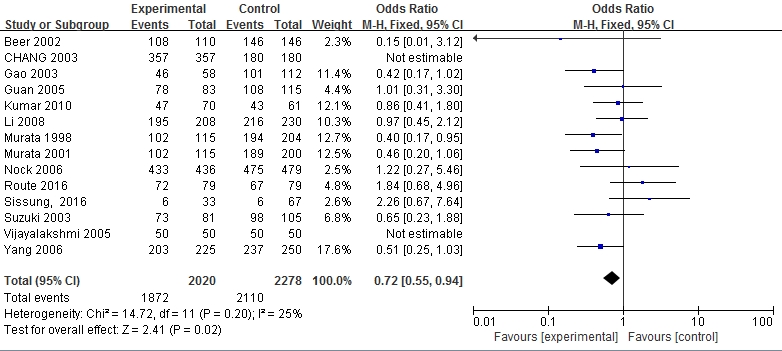

Supplement: Supplementary file 2 [file DataSheet2.zip › rs1048943/GG+GA VS AA森林图.jpg]

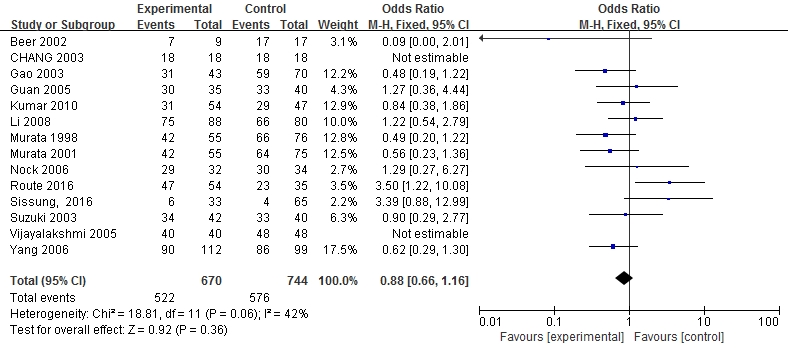

Supplement: Supplementary file 2 [file DataSheet2.zip › rs1048943/杂合 GA VS AA 森林图.jpg]

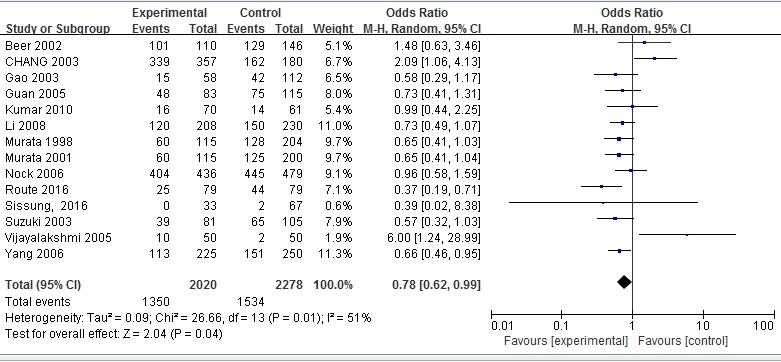

Supplement: Supplementary file 2 [file DataSheet2.zip › rs1048943/隐形模型 森林图.jpg]

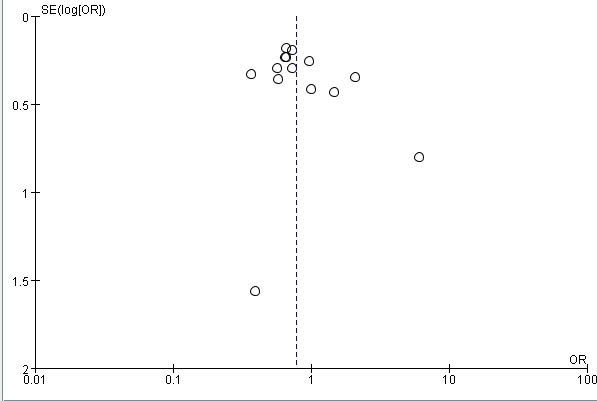

Supplement: Supplementary file 2 [file DataSheet2.zip › rs1048943/隐形模型 漏斗图.jpg]
